# The profile of Cyr61 expression data correlate to the skin inflammation in psoriasis

**DOI:** 10.1016/j.dib.2016.12.008

**Published:** 2016-12-15

**Authors:** Pinru Wu, Gang Ma, Ningli Li

**Affiliations:** a Shanghai Institute of Immunology, Institute of Medical science, Shanghai Jiao Tong University School of Medicine, Shanghai, China; b Department of Dermatology, Shanghai Ninth People׳s Hospital, Shanghai Jiao Tong University School of Medicine, Shanghai, China; c Department of Plastic and Reconstructive Surgery, Shanghai Ninth People׳s Hospital, Shanghai Jiao Tong University School of Medicine, Shanghai, China

**Keywords:** Psoriasis, Cysteine-rich 61, Inflammation

## Abstract

The data presented in this article are related to the research article entitled “Cyr61/CCN1 is involved in the pathogenesis of psoriasis vulgaris via promoting IL-8 production by keratinocytes in a JNK/NF-κB pathway” (Pinru Wu, Gang Ma, Xianjin Zhu, Ting Gu, Jie Zhang, Yue Sun, Hui Xu, Rongfen Huo, Beiqing Wang, Baihua Shen, Xiangdong Chen, Ningli Li, 2016) [1]. Cysteine-rich 61 (Cyr61/CCN1), a secreted extracellular matrix protein, is a novel proinflammatory factor. In this dataset skin samples from normal donors and psoriasis vulgaris patients were examined the expression of Cyr61 and IL-8 using immunohistochemistry. IMQ-induced psoriasis-like mice were treated with anti-Cyr61monoclonal antibodies (mAb).

**Specifications Table**

**Table:** 

Subject area	Health sciences
More specific subject area	Dermatology
Type of data	Figures, Text file
How data was acquired	NanoDrop ND-1000 Spectrophotometer, cDNA Synthesis Kit, Confocal laser scanning fluorescence microscopy
Data format	Raw and analyzed
Experimental factors	Psoriatic lesional skin of patients and IMQ-induced mice
Experimental features	Analyze the profile of Cyr61 expression in psoriatic lesion by Immunohistochemistry. qPCR was used for mRNA expression and protein expression by immunofluorescence studies.
Data source location	Department of Dermatology
	Shanghai Ninth People׳s Hospital, Shanghai JiaoTong University School of Medicine 639, Zhizaoju Road, Huangpu District, Shanghai, P. R. China, 200011
Data accessibility	The data are available with this article

**Value of the data**

• This data characterizes the distribution of Cyr61 expression in skin lesion of patients with psoriasis vulgaris.
• These data could be used for developing improved strategy in the treatment of psoriasis.

## 1 Data

**Fig. 1:**
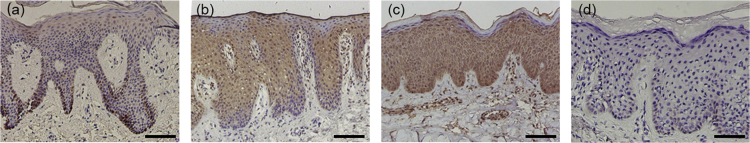
The occurrence and distribution of Cyr61 expression in skin lesion of patients with psoriasis vulgaris. The epidermal positive staining of Cyr61 varied from patient to patient: (a) darker staining in stratum basale, (b) darker staining in upper epidermal layers, (c) moderate staining in stratum spinosum, (d) isotype control. Bar 100 μm, Magnification ×100.

**Fig. 2:**
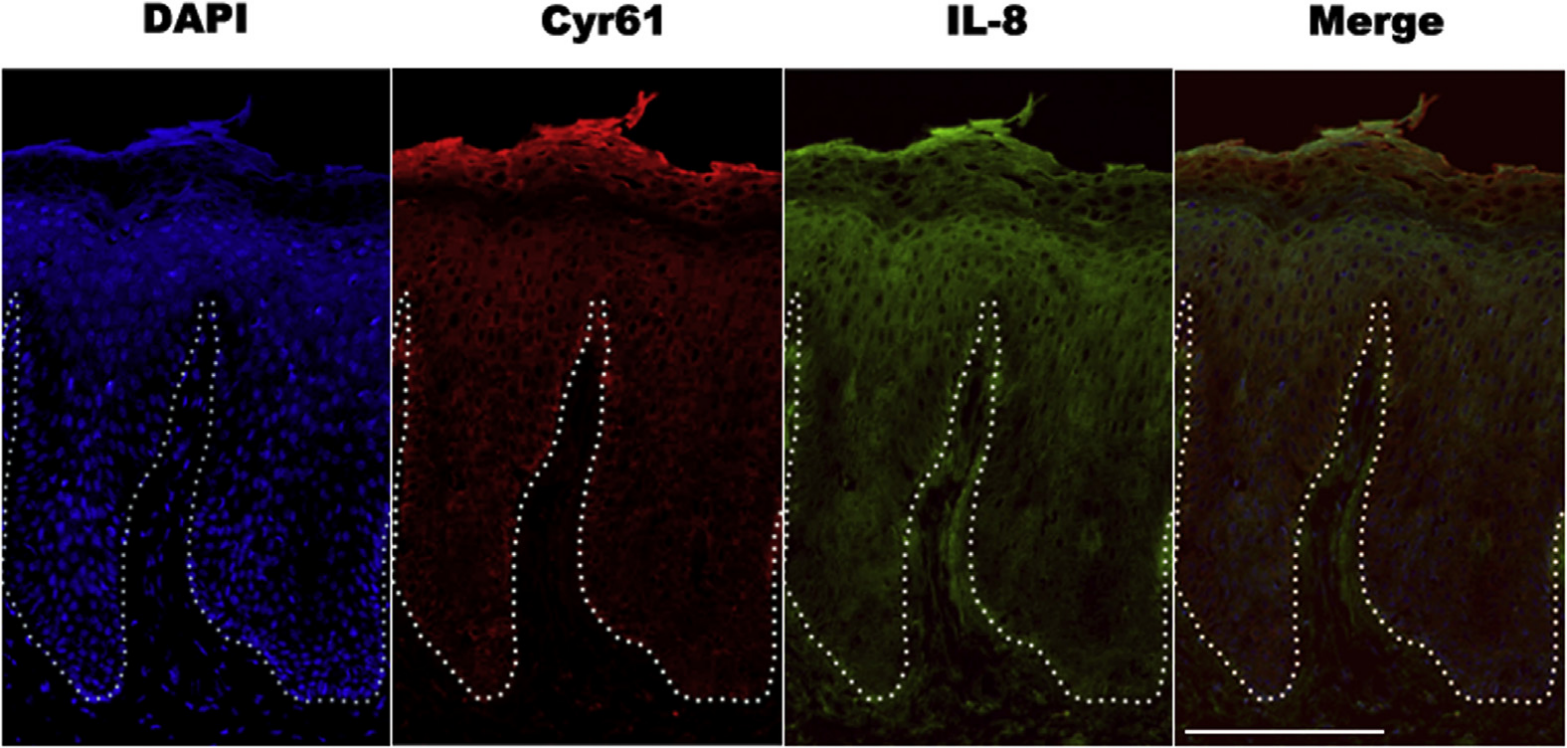
Representative immunofluorescent staining of Cyr61 and IL-8 in the lesional skin of psoriasis patient. Bar 100 μm.

**Fig. 3:**
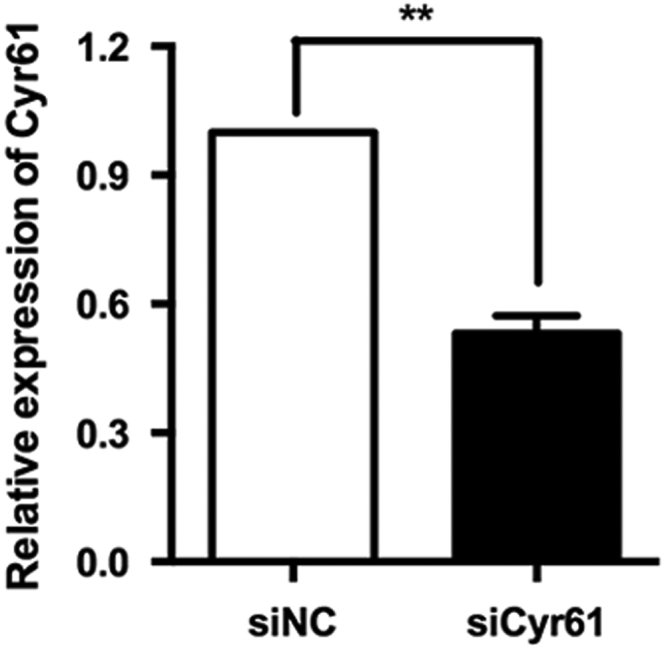
The identification of specific siRNA of Cyr61. The siRNA of Cyr61 was identified in HaCaT cells, which could reduce Cyr61 expression by about 50% at mRNA levels. **P<0.01.

**Fig. 4:**
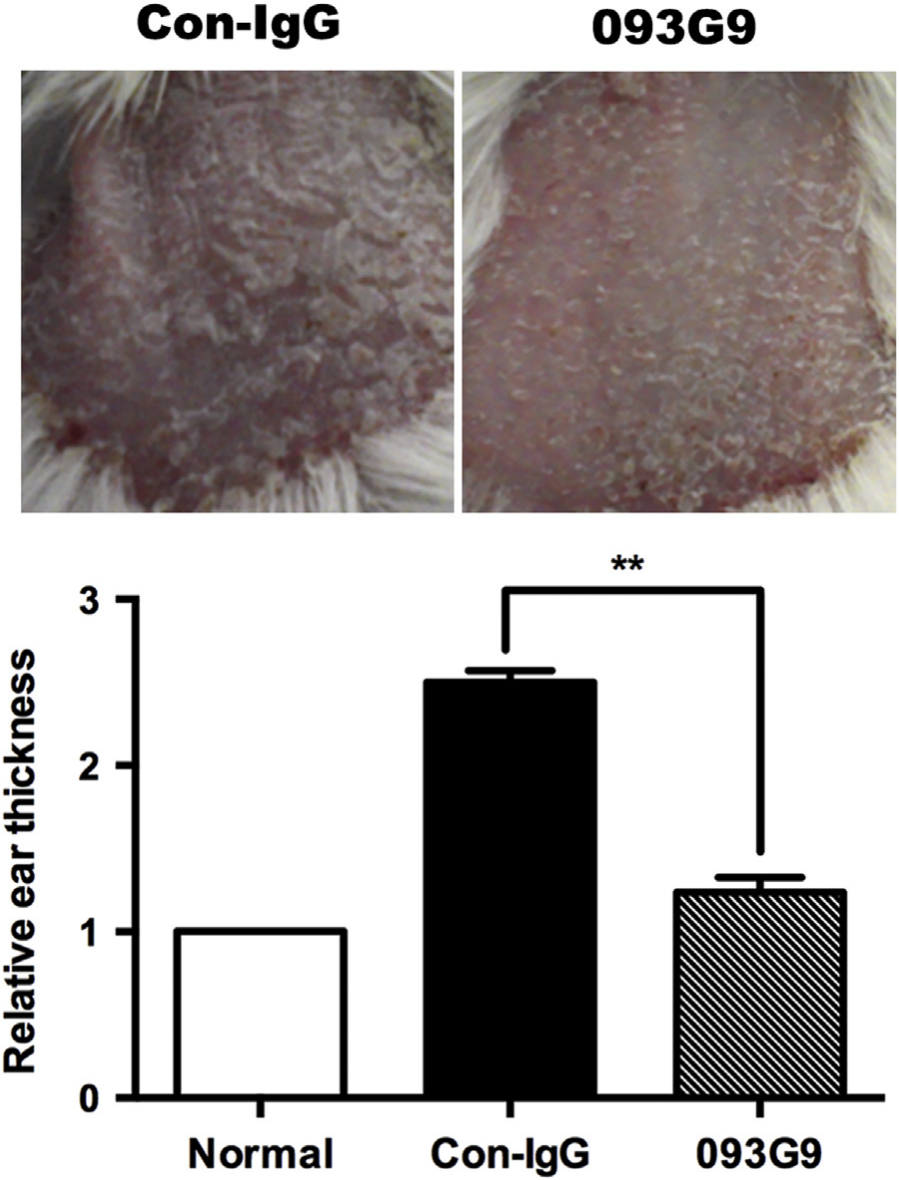
Symptoms in psoriasis-like mice treated with 093G9 were significantly inhibited by blocking Cyr61. The multilayer silvery white scales in the back skin and the incrassation of the ears were decreased after blocking Cyr61 expression. **P<0.01.

The dataset of this article provides information on the Cyr61 and IL-8 expression in skin lesion in psoriasis patients (Fig. 1, Fig. 2) and the change of the production of Cyr61 by specific siRNA *in vitro* and anti-Cyr61mAb *in viv*o (Fig. 3, Fig. 4).

## 2 Experimental design, materials and methods

### 2.1 Immunohistochemistry analysis of Cyr61 expression

Normal and lesional skin from human donors and mice were fixed in 4% paraformaldehyde, embedded in paraffin and sectioned. For immunohistochemistry, skin samples from patients were stained with anti-Cyr61 mAb at a concentration of 1:200 followed by HRP conjugated goat anti-mouse secondary antibody [1,2].

### 2.2 RNAi knockdown of gene expression

Cyr61, IL-1β and TNF-α small interfering RNA (siRNA, Table S2; see Supporting information) were designed and synthesized at Shanghai Genepharma (Shanghai, China) and gene knockdowns were performed as previously reported [3]. HaCaT cells were cultured in 24-well plates. A transfection mixture of siRNA oligonucleotides and Lipofectamine 2000 reagent (Invitrogen, Carlsbad, CA, USA) in serum-free medium was added to medium-aspirated cells for 4 h. Then, the medium was replaced with complete DMEM containing 10% fetal bovine serum for additional 24 h incubation.

### 2.3 Establishment and treatment of IMQ-induced psoriasis-like skin inflammation model in mice

For induction of the psoriasis-like skin inflammation model, mice received a daily topical dose of 62.5 mg of IMQ cream (5%) (Aldara, 3M Pharmaceutical, UK) on the shaved back and the right ear, representing a daily dose of 3.125 mg of the active compound. Control mice were treated similarly with vaseline (Vaseline Lanette cream, Fagron). Mice received intraperitoneal injections of 200 μg/day of either anti-Cyr61 mAb 093G9 or control IgG1 (Millipore, Billerica, MA, USA) 2 days after IMQ treatment. After16 days later mice were sacrificed and skin samples were collected and inspected [4].

## Funding sources

This work was funded by National Natural Science Foundation of China (81473682, 81402618), Excellent Young Doctor Foundation of Shanghai Ninth People׳s Hospital (201608), Education Ministry Research Fund for the Doctoral Program (20130073110003), Science and Technology Commission of Shanghai Municipality (13JC1402300) and Shanghai Cultivate Outstanding Young Teachers in Colleges and Universities Scientific Research Fund (JDY09062).

## Footnotes

Transparency document Transparency data associated with this article can be found in the online version at doi:10.1016/j.dib.2016.12.008.
